# Reduced Systolic Volume: Main Pathophysiological Mechanism in Patients with Orthostatic Intolerance?

**DOI:** 10.5935/abc.20160135

**Published:** 2016-10

**Authors:** Maria Zildany P. Távora-Mehta, Niraj Mehta, Adriano Magajevski, Larissa de Oliveira, Débora Lee Smith Maluf, Letícia Concato, Eduardo Doubrawa, Márcio Rogério Ortiz, Cláudio L. Pereira da Cunha

**Affiliations:** Hospital de Clínicas da Universidade Federal do Paraná, Curitiba, PR - Brazil

**Keywords:** Orthostatic Intolerance/physiopathology, Stroke Volume, Vascular Resistance, Postural Orthostatic Tachycardia Syndrome/physiopathology

## Abstract

**Background:**

Orthostatic intolerance patients' pathophysiological mechanism is still obscure, contributing to the difficulty in their clinical management.

**Objective:**

To investigate hemodynamic changes during tilt test in individuals with orthostatic intolerance symptoms, including syncope or near syncope.

**Methods:**

Sixty-one patients who underwent tilt test at - 70° in the phase without vasodilators were divided into two groups. For data analysis, only the first 20 minutes of tilting were considered. Group I was made up of 33 patients who had an increase of total peripheral vascular resistance (TPVR) during orthostatic position; and Group II was made up of 28 patients with a decrease in TPVR (characterizing insufficient peripheral vascular resistance). The control group consisted of 24 healthy asymptomatic individuals. Hemodynamic parameters were obtained by a non-invasive hemodynamic monitor in three different moments (supine position, tilt 10' and tilt 20') adjusted for age.

**Results:**

In the supine position, systolic volume (SV) was significantly reduced in both Group II and I in comparison to the control group, respectively (66.4 ±14.9 ml vs. 81.8±14.8 ml vs. 101.5±24.2 ml; p<0.05). TPVR, however, was higher in Group II in comparison to Group I and controls, respectively (1750.5± 442 dyne.s/cm^5^ vs.1424±404 dyne.s/cm^5^ vs. 974.4±230 dyne.s/cm^5^; p<0.05). In the orthostatic position, at 10', there was repetition of findings, with lower absolute values of SV compared to controls (64.1±14.0 ml vs 65.5±11.3 ml vs 82.8±15.6 ml; p<0.05). TPVR, on the other hand, showed a relative drop in Group II, in comparison to Group I.

**Conclusion:**

Reduced SV was consistently observed in the groups of patients with orthostatic intolerance in comparison to the control group. Two different responses to tilt test were observed: one group with elevated TPVR and another with a relative drop in TPVR, possibly suggesting a more severe failure of compensation mechanisms.

## Introduction

Orthostatic intolerance syndrome corresponds to a heterogenic group of hemodynamic regulation disorder and is defined by the appearance of various and unspecific symptoms that may be related to cerebral hypoperfusion in orthostasis.^1-2^ Patients frequently develop symptoms of dizziness, visual turbidity, fatigue, nausea, near syncope, or syncope during prolonged orthostasis.^1,3^ Clinical management is complex, largely due to lack of knowledge about pathophysiological mechanisms. Previous studies have shown that neurohumoral changes,^4^ deconditioning,^5^ and hypovolemia^6^ may be involved.

Chronic orthostatic intolerance occurs in some individuals with postural orthostatic tachycardia syndrome, neuromediated syncope, and in some clinical situations that occur with postural hypotension. Blood pressure (BP) needs to be adequately maintained, not only during rest, but also during several daily activities, such as: physical exercise, mental stress, and digestion. The inability to maintain it, especially in orthostasis, may result in reduced systemic perfusion, especially cerebral, due to its anatomical location above the heart.^7,8^

Many patients do not present alterations in heart rate (HR) or BP during orthostatic symptoms. This suggests that there may be pathophysiological alterations in different degrees of severity, which reflects the wide range of unspecific symptoms. Morevoer, many patients with frequent neuromediated syncope episodes also present with orthostatic symptoms between syncope episodes.^8^ The absence of clear alterations in BP and HR may represent altered mechanisms of cerebral perfusion autoregulation. If there are no evident BP or HR alterations, there still may be hemodynamic alterations that precede pressure drop, such as reduced systolic volume (SV) and altered peripheral vascular resistance. Therefore, it is necessary to identify different response patterns to the tilt test, with the objective of identifying possible pathophysiological mechanisms. Knowledge of the pathophysiological mechanism involved in these patients may help to manage them in a clinical context.

The aim of the present study is to evaluate the hemodynamic parameters, such as peripheral vascular resistance and SV responses, when individuals with orthostatic intolerance symptoms transition into orthostasis. These symptoms include syncope or near syncope of obscure etiology (no evidence of arrhythmia or ventricular dysfunction and without orthostatic tachycardia or hemodynamic collapse during tilt test at 70°, free of medication), and the individuals were compared to a control group made up of healthy individuals (asymptomatic).

## Methods

### Studied population

Patients were recruited at the syncope and autonomic disorders laboratory at *Hospital de Clínicas da Universidade Federal do Paraná* and from the Cardiac Electrophysiology Service of Paraná, Brazil.

This is a retrospective case-control study. A total of 61 consecutive patients were included from a total of 117 who were referred in the period between February 2013 and May 2014, for the realization of tilt tests for orthostatic intolerance symptoms, including syncope and/or near syncope. Syncope or near syncope symptoms were recurring and related to changes in position, or, vertical, seating or standing positions. In the studied sample, there were no patients with situational syncope associated with physical trauma, accident, or physical exercise. All patients presented a negative response during 20 minutes of tilting in the phase free of medications for vasovagal reaction and postural orthostatic tachycardia syndrome (POTS).

The patients who were referred to the tilt test were already under previous investigation with a 24-hour Holter, echocardiogram, scintigraphy and/or catheterization. Of these 117 patients, 56 were excluded for the following reasons: under 16 years old (2 patients); documented ventricular dysfunction (1); documented obstructive or sustained ventricular coronary artery disease (7); stroke or other confirmed neurological disease (44); debilitating systemic disease (2), or reduced life expectancy (<1 year) and individuals with pure autonomic failure or Parkinson's disease. The studied population did not present with other diagnosed comorbidities other than hypertension, and two of the included patients had diabetes with no target-organ lesion.

Therefore, the present study is about the evaluation of 61 patients with orthostatic intolerance, where the differential diagnoses of syncope and near syncope were excluded.

The control group consisted of 24 healthy asymptomatic individuals, between 17 and 39 years of age, whose voluntary participation was accepted upon signature of the free consent form.

The present study was duly approved by the Ethics Committee of local research.

### Complete standard protocol for the tilt test:

All included patients (61) underwent a tilt test at 70°, after six hours of fasting, in the 20-minute protocol free of drugs (period analysed in our study). If results were negative on this period, patients were sensitized with 0.4 mg of sublingual nitroglycerin and kept on inclination for another period of up to 15 minutes, except when systolic blood pressure (SBP) was under 90 mmHg. In that case, with SBP under 90 mmHg after 20 minutes, these patients were kept on inclination for another 10 minutes, without drugs. The tilt test was interrupted, at any moment during the exam, in case of a vasovagal reaction, characterized by a drop in HR and/or BP associated to symptoms of syncope or near syncope. Room temperature was kept between 23° and 25°C. Beta-blockers were suspended for at least 5 half-lives before the exams. Diuretics were suspended at least 72 hours before the exam.

The exams were performed with a hemodynamic monitor (Task Force Monitor® CNSystems Medizintchnik AG Austria, 2008) with continuing measurement of BP, HR and SV through bioimpedance. A set of electrodes, constituted by four electrocardiogram electrodes and three band electrodes and one neutral electrode especially developed for bioimpedance derivation, were fixated to the patients. Peripheral vascular resistance (TPVR) was calculated by the device with the BP formula, in which BP = HR x SV x TPVR, where TPVR = BP/SV x HR, and cardiac debit (CD) was also calculated by the device according to the formula: CD = HR x SV.

During the exam, mean values of hemodynamic parameters were analysed in five traditional periods, programmed by the equipment manufacturer: supine position (S); 0 to 5 (tilt 5'); 5 to 10 (tilt 10'); 10 to 15 (tilt 15') and 15 to 20 (tilt 20') minutes of inclination.

### Hemodynamic parameter analysis of the present study

In the 61 patients included in this study, hemodynamic parameters were evaluated only during basal inclination period - 20-minute drug-free period. Of the measured hemodynamic parameters, only the analyses of the data in 3 distinct times are part of this study, because they reflect relevant moments of the orthostasis period and because they simplify the description of the findings. Therefore, the groups were compared in relation to the results on: supine position (S), tilt 10' and tilt 20'. That is, the mean values at rest in the supine position, mean values from 5 to 10 minutes (of inclination) and mean values from 15 to 20 minutes (of inclination). Moreover, differences from one moment to another were also analysed (deltas between position S, tilt 10' and tilt 20').

We first observed that there were two distinct responses to the inclination period: one group had a rise in TPVR, and the other showed a drop. Thus, due to the discrepant responses, the analysis was done by separating these two groups.

**Figure 1:**
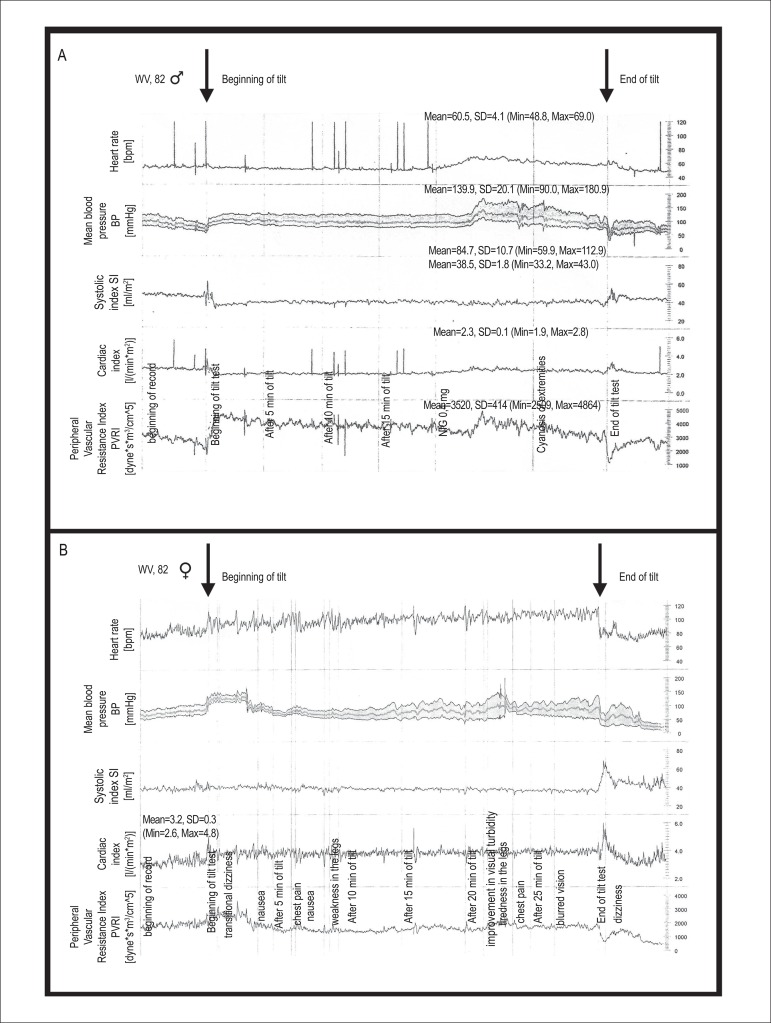
Non-invasive hemodynamic index monitor records. A) Patient in Group I – observe the compensatory elevation of TPVR index during inclination. B) Patient in Group II – observe the unexpected drop in TPVR during inclination.

Thus, all patients were divided into two groups according to TPVR response during the tilt test in the drug-free phase: Group I (33 patients), corresponds to the patients who presented an increase in TPVR in orthostasis (compared to the supine position), and who maintained this increase for the entire 20 minutes of drug-free inclination. Group II (28 patients) corresponds to those who presented, in one of the studied intervals during the inclination period (tilt 10' or tilt 20'), mean values that were lower than those observed in the supine position. These patients were considered to have TPVR insufficiency (Figure 1) because they were not able to increase or maintain an increase in TPVR in the 20-minute drug-free inclination period. Both groups were compared to a control group of asymptomatic healthy individuals.

### Statistical analysis

Initially, the groups were compared in relation to the results at each moment of evaluation (S, tilt 10' and tilt 20') and in relation to the differences that occurred from one moment to the other (supine-10', supine-20' and 10'-20') - deltas. To that end, we tested the null hypothesis that the means were equal in all three groups, versus the alternative hypothesis that at least one group had a different mean than the others. If there were a significant difference among the groups, these were compared two by two. Results were adjusted for age. We used Student T test or Mann-Whitney for different samples (p < 0.05).

Results obtained were described by means, medians, minimum values, maximum values and standard deviations. For the comparison of evaluation moments (supine, tilti10' and tilt 20'), within each group, we used the analysis of variance model with repeated measures. To compare the groups in relation to the moment of evaluation and differences between the moments of evaluation (tilt 10' - S, tilt 20' - S e tilt 20' - tilt 10'), we considered the analysis of covariance model (ANCOVA), including age as a covariable. For multiple comparisons (post hoc), we used the LSD test (least significant test). In relation to variables of age, height, weight and body surface, the groups were compared using the analysis of variance model (ANOVA) with one factor. Values of p < 0.05 indicated statistical significance.

For each group, at each moment of evaluation, we tested the null hypothesis that there is no association between SV and TPVR, versus the alternative hypothesis that an association is present. Estimated values of the Pearson correlation coefficient and p values of statistical tests are presented in tables and correlation graphs. Data were analysed with the computer program SPSS v.20.0.

Group homogeneity evaluation was carried out in relation to age, weight, height, body mass index, and body surface (Table 1).

**Table 1:** Group homogeneity evaluation in relation to age, weight, height, body mass index and body surface

Variable	Group	Nº	Mean	Median	Minimum	Maximum	Standard Deviation	p value*
	I	33	56.2	58.0	18.7	90.2	16.9	
	II	28	57.4	61.6	16.2	86.9	22.6	
	Control	24	27.6	28.5	17.0	39.0	6.2	<0.001
Height (cm)	I	33	165.5	165.0	150.0	182.0	7.8	
	II	28	168.4	168.5	151.0	190.0	8.9	
	Control	24	170.2	169.0	152.0	192.0	10.9	0.161
Weight (kg)	I	33	70.6	71.0	47.0	110.0	12.6	
	II	28	75.3	75.0	50.0	109.0	15.4	
	Control	24	71.5	68.5	49.0	118.0	15.5	0.433
Body mass index	I	33	25.7	26.1	18.4	33.2	3.4	
(kg/m^2^)	II	28	26.6	26.3	18.8	36.0	5.1	
	Control	24	24.4	24.2	20.1	32.0	3.0	0.157
Body surface (cm^2^)	I	33	1778.0	1765.0	1462.0	2304.0	182.2	
	II	28	1845.6	1853.0	1467.0	2283.0	196.5	
	Control	24	1739.3	1757.0	642.0	2468.0	361.0	0.298

* ANOVA with p<0.05 factor

## Results

Concerning the studied hemodynamic parameters, the obtained data in the supine position during inclination (tilt 10' and tilt 20') are found in Table 2.

**Table 2:** Hemodynamic parameters (mean SD) in the supine position and during tilt 10' and tilt 20' in the drug-free phase

	Group I (I)	Group II (II)	Control (C)	p value (IxIIxC)	p value (IxII)	p value (I x C)	p value (II x C)
HR Supine* (bpm) HR (tilt 10')* (tilt 20')*	69.5±11 78.3±13.0 81.6±13.5	70.0±11.7 80.3±15.2 84.7±15.9	70.4±9.7 87.2±11.9 84.7±15.9	0.63 0.75 0.51	- - -	- - -	- - -
MBP Supine* (mmHg) MBP (tilt 10')* (tilt 20')*	96.6±11.4 105.6±10.6 103.4±12.2	97.7±15.7 99.4±15.3 94.7±12.5	84±8.7 102.1±10.0 98.9±9.1	0.13 0.13 0.01	- - 0,005	- - 0,15	- - 0,19
SV Supine* (ml) SV (tilt 10')* (tilt 20')*	81.8±14.8 65.5±11.3 61.4±8.6	66.4±14.9 64.1±14.0 62.4±14.4	101.5±24.2 82.8±15.6 79.6±16.6	<0.001 0.003 0.007	0.001 0.705 0.76	<0.001 <0.001 <0.001	<0.001 <0,001 <0.001
CD Supine* (l/min) CD (tilt 10')* (tilt 20') *	5.71±1.57 5.06±1.0 5.03±1.0	4.53±0.81 5.02±0.84 5.11±0.92	6.95±1.56 7.13±1.3 7.33±1.43	<0.001 <0.001 <0.001	<0.001 0.86 0.75	<0.001 <0.001 <0.001	<0.001 <0.001 <0.001
TPVR Supine* ┼ TPVR (tilt 10')* (tilt 20') *	1424±404 1725±441 1704±466	1750±442 1576±324 1482±323	974.4±230 1155±222 1089±207	<0.001 0.003 0.001	<0.001 0.08 0.011	<0.001 <0.001 <0.001	<0.001 <0,001 <0.001

* p value adjusted for age (ANCOVA)

┼ TPVR in dyne.seg/cm^5^

HR: heart rate; MBP: mean blood pressure; SV: systolic volume; CD: cardiac debit; TPVR: total peripheral vascular resistance.

**Figure 2:**
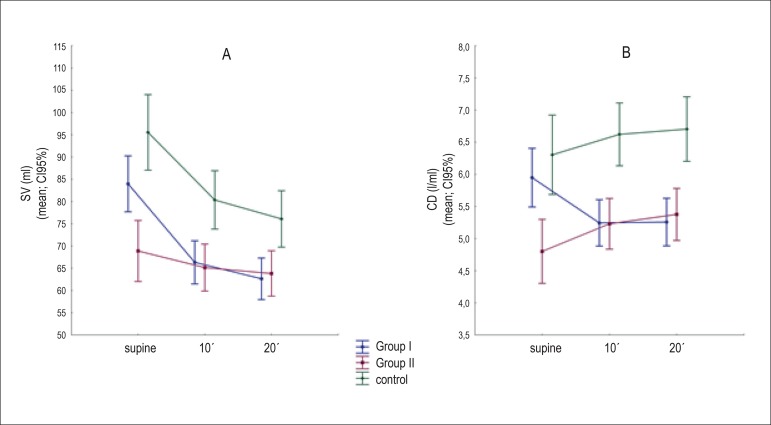
A and B) Means and confidence intervals of 95% for SV and CD means adjusted for age: comparison between the groups at each moment of analysis (supine, tilt 10' and tilt 20'). SV: systolic volume; CD: cardiac debit.

**Figure 3:**
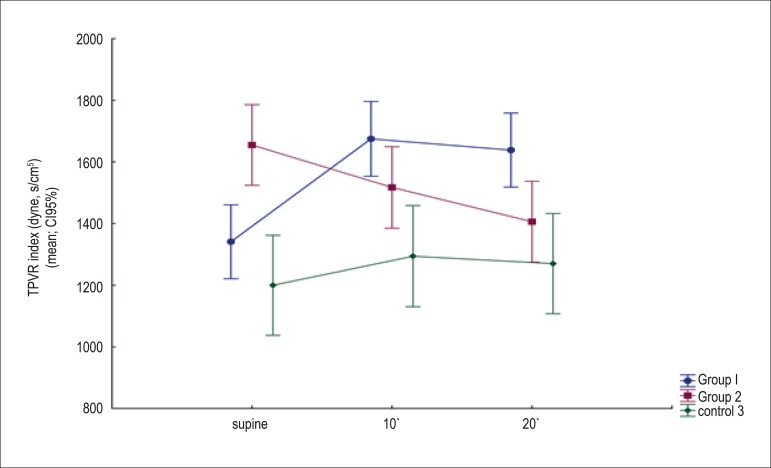
Means and confidence intervals of 95% for TPVR means adjusted for age: comparison between the groups at each moment of analysis (supine, tilt 10' and tilt 20').

**Figure 4:**
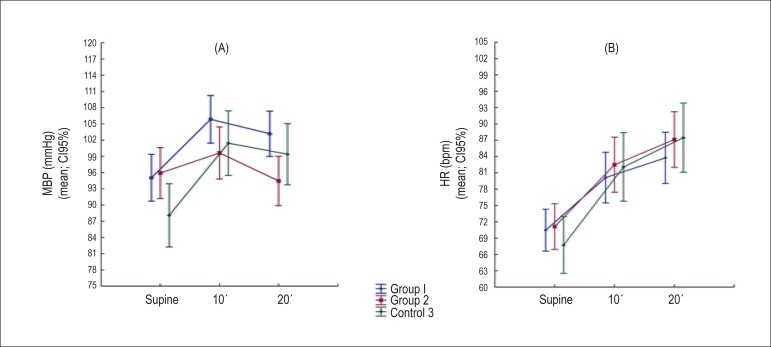
A and B) Means and confidence intervals of MBP and HR means adjusted for age: comparison between the groups at each moment of analysis (supine, tilt 10' and tilt 20'). MBP: mean blood pressure; HR: heart rate.

In the supine position, we observed that SV and CD were significantly reduced in Group II patients in relation to those in Group I, and patients in Group I showed reduced SV and CD in comparison to individuals in the control group (Figure 2). The opposite was observed in relation to TPVR, which was more elevated in Group II, in comparison to Group I; and in Group I, TPVR was more elevated than in the control group (Figure 3). Thus, we observed an inverse correlation between SV and TPVR in all three groups. On the other hand, no significant difference was observed between the groups in relation to mean BP and HR in the supine position (Figure 4).

In orthostasis, we saw a progressive reduction of TPVR in Group II, whereas, in Group I, we saw an elevation of TPVR (Table 2). We observed that, between these two groups, there was no difference in the absolute value of TPVR in the period of tilt 10'. At this moment, we also did not observe differences between Group I and Group II in any of the other studied parameters, with significant differences in TPVR being observed again in the period of 20'. Contrary to what was seen in the supine position, at this moment (tilt 20'), TPVR was lower in Group II (Figure 3), suggesting more severe failure of compensatory mechanisms of hemodynamic regulation.

When compared to the control group, both Group I and Group II showed, at every moment during the exam, significantly lower SV and CD and significantly higher TPVR.

BP did not differ between the groups, except during the 20' tilt, due to a progressive reduction in TPVR observed in orthostasis in Group II (Figure 4-a). HR, in turn, did not present significant differences among all three groups at any analysed moment (Figure 4-b).

In the analyses of differences (Δ, delta), in the period between supine and tilti10', TPVR, increased in Group I and in controls, making a positive delta (compensatory natural response). On the other hand, in Group II, TPVR decreased, resulting in a negative delta, which characterizes peripheral vascular resistance insufficiency in the group. Since SV in Group II was already more severely reduced in the supine position, there was no severe drop of SV in orthostasis. Therefore, this group showed a significantly smaller decrease in SV in relation to Group II and controls. With regards to BP, we observed that, in the last evaluated period (tilt 20'), the difference of mean BPs in relation to the supine position in Group II was negative (suggesting failure of compensatory hemodynamic mechanisms) (Table 3).

**Table 3:** Difference of hemodynamic parameters (mean ± SD) between the periods of tilt' 10' and supine position (∆ 10'-S) and between the period tilt 20' and supine position (∆ 20'-S)

	Group I	Group II	Control (C)	(I x II) p	(I x C) p	(II x C) p
HR (bpm) (∆10'-S) (∆ 20'-S)*	8.8±7.2 12.1±7.6	10.3±7.8 14.7±9.4	16.8±6.6 22.8±8.3	NS 0.38	NS <0.001	NS 0.0001
SV (ml) (∆10'-S)* (∆ 20'-S)*	-16.3±12.1 -20.4±10.1	-2.2±11.2 -4.0±11.2	-18.8±18.4 -22.0±18.2	<0.001 <0.001	0.50 0.66	<0.001 <0.001
TPVR ┼(∆10'-S)* (∆ 20'-S)	301±218 280±176	-174±328 -268±266	180±260 115±190	<0.001 <0.001	0.09 0.005	<0.001 <0.001
MBP (mmHg) (∆10'-S)* (∆ 20'-S)*	9.1±8.6 6.8±10.6	1.7±15.4 -3.0±-39.9	18.1±9.4 14.9±4.9	0.010 0.001	0.003 0.008	<0.001 <0.001

* p value adjusted for age (ANCOVA).

┼ TPVR in dyne.seg/cm^5^

HR: heart rate; MBP: mean blood pressure; SV: systolic volume; TPVR: total peripheral vascular resistance.

**Figure 5:**
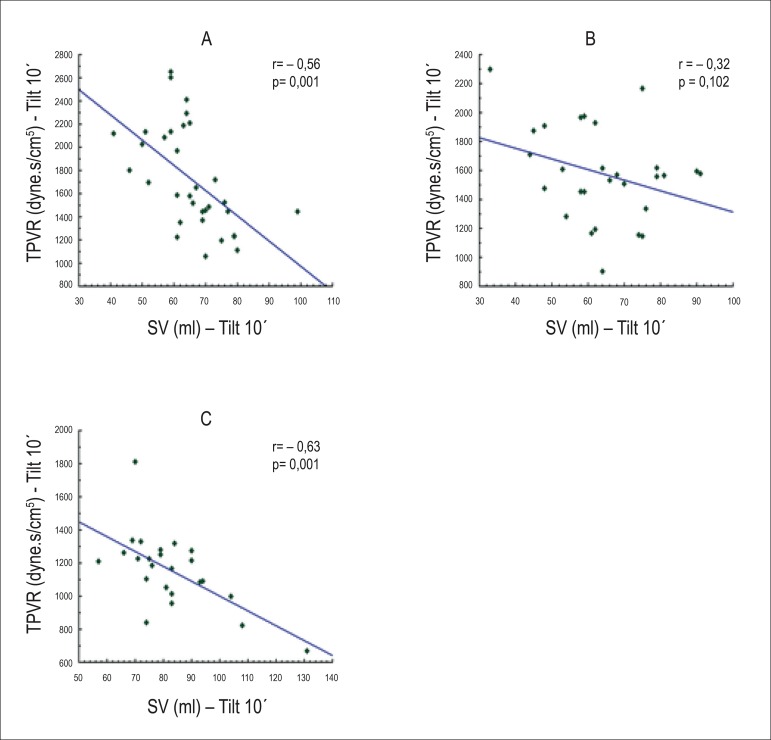
a (Group I), b (Group II) and c (Control group). Scatter diagrams for SV and TPVR at 10' during tilt test; r: Pearson correlation coefficient. SV: systolic volume;TPVR: total peripheral vascular resistance.

In the correlation analysis between SV and TPVR, we observed a significant inverse correlation in the three groups in the supine position. However, in the period of tilt 10', the negative correlation disappeared in Group II, because vascular resistance was reduced in othostasis (Figure 5).

We would like to highlight three points:

1. The studied patients, with orthostatic intolerance symptoms, including syncope and/or near syncope, presented with significantly lower SV and CD and significantly higher TPVR, in both the supine and orthostatic positions when compared to the control group, and did not show differences in relation to BP or HR in either position.
2. Among the studied patients, 46% presented TPVR insufficiency during the tilt test. In the supine position, they presented more reduced SV and CD and more elevated TPVR in relation to the other studied patients.
3. At tilt 10', in turn, we observed a loss of the inverse correlation between SV and TPVR in Group I, due to the failure of compensatory mechanisms represented by the drop in TPVR.

## Discussion

The main finding of this study was the observation of a reduced SV in the group of patients with orthostatic intolerance symptoms in relation to the control group, in both the supine and the orthostatic positions.

It has been suggested that all forms of orthostatic intolerance may be a result of central hypovolemia, even without tachycardia.^2^ Central hypovolemia,^9-11^ and reduced SV^12,13^ have been consistent findings in patients with POTS in othostasis. In a preliminary study, Távora-Mehta et al. observed that patients with orthostatic intolerance symptoms, even without POTS, presented similarly reduced SV values, when SV was corrected for body surface.^14^ In the present study, the comparison with the control group reinforces previous findings. Therefore, a reduced SV in individuals with orthostatic intolerance symptoms is a finding that seems consistent, even in those who do not develop orthostatic tachycardia or hemodynamic collapse during the tilt test.

In normal individuals reported in literature, the main findings after approximately 5 minutes in orthostasis (in comparison to the supine position), are the decrease of about 30% of thoracic blood volume and SV, HR increase of 15-30%, accompanied by CD reduction of about 20%,^15-17^ similarly to what was observed in the control group in the present study, except in regards to CD. In the present study, the observed that SV decrease in othostasis was appropriately compensated by the increase in HR, as to avoid a decrease in CD.

In order to maintain BP and cerebral perfusion regardless of gravity effects, a series of regulatory or reflex cardiac mechanisms are activated. To that end, HR, SV (consequently CD) and TPVR are modulated, having BP as a controlled variable.^18,19^ In the present study, when going into orthostasis, we observed, in all patients, an increase in BP in the first 10 minutes, except in Group II. In Group II, we observed a mean BP that was lower than in the supine position only at tilt 20', when it became significantly lower in relation to the other groups, suggesting failure of compensatory mechanisms of natural elevation of peripheral resistance.

In the control group, SV was significantly higher. On the other hand, in the patient groups, TPVR was higher. Considering the following formula: BP = CD x TPVR, where CD = HR x SV, with similar HR between controls and patients, BP becomes more dependent on volume in the control group of healthy individuals. In the group of patients with othostatic symptoms, however, maintenance of BP was more dependent on vascular resistance. Thus, it would not suffice to reduce TPVR in the supine position to treat such patients, but, concomitantly, provide an elevation of SV. Qi Fu et al.^13^ showed that physical activity is one of the ways to increase SV in POTS patients who had reduced SV. Recently, intravenous hydration (with 1 to 2 liters/day for 3-7 days a week) showed clinical improvement in patients with refractory orthostatic intolerance.^20^

The systems responsible for cardiovascular regulation control include: naurocardiovascular system, humoral system (renin-angiotensin and vasopressin), capillary system and renal system (aldosterone and antidiuretic hormone). BP hemodynamic stabililty, in the initial phase of othostasis (30 seconds to 2 minutes) is obtained mainly by the neurocardiovascular system.^15,18^ Muscle sympathetic nerve activity increases with the change in position, resulting in baroreflex-mediated vasoconstriction.^21^ Sympathetic baroreflex sensitivity increases during posture change, but remains unaltered during orthostasis. Thus, there is a positive relation between TPVR and muscle sympathetic nerve activity, suggesting that the control of the vasomotor sympathetic nerve is still important in at least 45 minutes of othostasis.^22^ It has also been observed that the increase in muscle sympathetic nerve activity is associated to a reduction in SV, with an inverse relation between the two.^22^

In the supine position, in the present study, we observed, in all groups, a significant inverse correlation between TPVR and SV. In Group II, this relation becomes even more evident.

In orthostasis, in the peripheral vascular resistance insufficiency group (Group II), there was a loss of the inverse relation between SV and TPVR in the first 10 minutes of inclination, because TPVR did not show the expected increase proportional to SV reduction, showing that this group, other than presenting a reduced SV, did not show the ability to appropriately compensate for TPVR. The data we found suggest that a more elevated TPVR in the supine position, in Group II in relation to other patients, may work as one of the compensation mechanisms to allow more tolerance to orthostasis.

In literature, studies with dysautonomia patients who present with severe hemodynamic repercussions in orthostasis, manifest insufficiency to increase TPVR and pronounced reduction in CD, when compared to healthy individuals. This is due to an increase in venous capacitance and inappropriate chronotropic response.^23-25^ However, the patients in the present study did not present hemodynamic repercussion despite the presence of peripheral vascular resistance insufficiency in orthostasis. We observed a discreet reduction of SV in orthostasis, so that the SV delta, in this group, was significantly lower than in other groups.

Reduced venous compliance, in this group, was another factor that influenced hemodynamic stability, since HR elevation was enough to maintain BP, despite TPVR reduction during orthostasis. As part of the tilt test protocol, it is necessary to stop the use of beta-blockers for at least 5 half-lives. It is possible that with during use of beta-blockers, many of these patients present more hemodynamic repercussions because the compensation mechanism to increase HR is blocked.

The compromised ability to increase vascular resistance in orthostasis caused by abnormalities in the autonomic nervous system is the main cause of postural hypotension or syncope in patients with several primary disorders (pure autonomic failure, Parkinson's disease) and secondary disorders (diabetes mellitus, uremia).^24^ In these patients with adrenergic failure, we can observe, during the tilt test, a progressive reduction of BP and pulse pressure, and HR response may be attenuated or increased when heart innervation is preserved. An increase in HR and BP fluctuations indicates that compensatory mechanisms are still intact, but also suggests an abnormality, because it indicates that the system is overloaded (overactivated).^25^ A reduction in vascular alpha-adrenergic sensitivity has been observed in patients with orthostatic intolerance symptoms, with POTS patients having the most compromised response, and in whom we observed a higher inability for TPVR elevation during orthostatic stress.^26^ Decreased sensitivity of alpha-adrenergic vascular receptors during orthostatic stress may be one of the hypotheses for alterations in TPVR response observed in Group II patients in this study.

Even though the patients were kept hemodynamically stable for a period of 20 minutes in orthostasis, we observed, in Group II patients, a significantly lower mean BP and TPVR in relation to Group I at tilt 20'. If this decrease is sustained for long periods of orthostasis, it may result in a greater reduction of BP. It is possible that cerebral and peripheral perfusion be compromised, even in hemodynamic stability (with low BP in the lower threshold for imminent syncope).

### Study limitations

In this study, due its retrospective characteristic, the quantification of syncope and near syncope symptoms were compromised. We did not perform dosages of serum catecholamine, since the main objective was to evaluate hemodynamic alterations. Monitoring of hemodynamic parameters was performed through a non-invasive method, validated in previous studies.^27^

The number of patients was limited and measurements of hemodynamic parameters were not corrected for body surface, since it was similar among the groups.

Another point to consider is the difference in age group among the studied groups and controls. Because young individuals may present a different hemodynamic response, we were careful to adjust data for age (ANCOVA, with age as a covariable).

Four patients were on medications with potential vasodilator effects (ACE inhibitors and calcium channel blocker). These drugs were not suspended, but these patients were part of Group I, where there was an expected response of TPVR elevation.

## Conclusions

Reduced SV was consistently observed in groups of patients with orthostatic intolerance, when compared to controls.

Among the studied patients, 46% presented with peripheral vascular resistance insufficiency in orthostasis during tilt test. In these patients, in the supine position, we saw greater reduction in SV and CD and a more elevated TPVR in relation to the other patients in the study. At 10 minutes of tilt, in this group, we observed loss of inverse correlation between SV and TPVE, while, in other patients, it was maintained throughout the entire exam.

### Clinical implications

Patients with non-specific symptoms of dizziness, dyspnea, and chest discomfort may have orthostatic intolerance, without presenting classical vasovagal syncope or measurable orthostatic hypotension. Identification that SV may play an important role in this condition suggests that non-pharmacological measures to increase it (i.e., increased fluid intake, regular exercise) may help treat these patients.
